# Clinical investigation of nosocomial infections in adult patients after cardiac surgery

**DOI:** 10.1097/MD.0000000000024162

**Published:** 2021-01-29

**Authors:** Zhengqin Liu, Xiquan Zhang, Qian Zhai

**Affiliations:** Qilu Hospital Affiliated to Shandong University, Jinan, Shandong, China.

**Keywords:** fungi, G- bacilli, G+ cocci, multiple drug resistance, nosocomial infections, risk factors

## Abstract

Nosocomial infections (NI) are common complications after cardiac surgery. To date, there have been few manuscripts investigating NI in the intensive care unit after cardiac surgery. Our study was designed to investigate the characteristics of the distribution of pathogenic bacteria, antibiotic resistance and risk factors for NI.

A total of 1360 patients received standard postoperative care, including antibiotic prophylaxis. Microbiological examinations of sputum, blood, catheter tips and excrement were performed as clinically indicated to isolate pathogens. Thirty potential associated variables were collected and compared between the 2 different groups according to the development of NI using univariate and multivariate analyses.

Eighty-nine patients (6.54%) acquired a microbiologically documented NI. There was a significant difference in mortality between the 2 groups with or without postoperative NI (23.60% vs 2.28%, *P* < .00). A total of 98 pathogens (73.13%) were isolated from sputum, 32 pathogens (23.88%) from blood and only 1 (0.75%) from urine. Three (2.24%) surgical site infections were detected, including 2 superficial surgical site infections and 1 mediastinitis. The most common pathogens were Gram-negative bacteria (78.36%), followed by Gram-positive bacteria (14.93%) and fungi (6.71%). The major pathogenic species had different levels of drug resistance, and most of them exhibited multidrug resistance. Six out of thirty variables were identified as independent risk factors for the development of NI, namely, duration of surgery, low cardiac output syndrome, continuous veno-venous hemofiltration, mechanical ventilation time, reintubation and tracheostomy.

We analyzed the characteristics of the distribution of pathogens, antibiotic resistance and risk factors for NI in our center and provided some suggestions for clinical practice. In addition to antibiotic treatment, avoidance of risk factors and aggressive infection control measures may be crucial to stop or prevent outbreaks.

## 1 Introduction

Nosocomial infections (NI) include all infections acquired between 48 hour after hospital admission and 2 days after hospital discharge. The cardiac surgical intensive care unit (ICU) is a special ward with a higher incidence of NI and usage rate of antibiotics, owing to the severity of illnesses, complexity of surgeries and common use of invasive devices (endotracheal tubes, central venous catheters, peripheral arterial catheters and urinary catheters).^[1]^ NI are associated with increased morbidity and mortality, as well as increased length of ICU stay and healthcare costs.^[2,3]^ Furthermore, the burden of antimicrobial resistance in the ICU is increasing, which has been attributed to the increased difficulty of clinical treatment and hospital control of NI. Therefore, initial antibiotic treatments should be updated to pathogen-specific treatments as soon as possible. The aims of our study were to investigate the characteristics of distribution of pathogenic bacteria, antibiotic resistance and risk factors for NI.

## 2 Materials and methods

### 2.1 Study population and design

From January 2018 to December 2018, 1381 patients (≥18 years old) undergoing open heart surgery were transferred to our cardiac surgical ICU. All patients were eligible for the investigation, except for those who died within 24 hour postoperatively (n = 21). Since it was a retrospective observational study, ethical approval was not necessary. All patients received standard postoperative care that complied with published guidelines.^[4,5]^ Antibiotic prophylaxis was a single administration of second-generation cephalosporin. Body temperature was recorded every 6 hour routinely and anytime when necessary. Hematologic tests and chest radiographs were performed regularly. Microbiological examinations of the sputum, blood, catheter tips and excrement of these patients were performed as clinically indicated to isolate pathogens according to the criteria of the Clinical and Laboratory Standards Institute. One pathogen cultured positively multiple times in the same patient within 1 week was regarded as a single infection. All enrolled patients were divided into 2 different groups: the NI group and non-NI group, according to the development of NI or not. Thirty potential related perioperative parameters, including age, gender, BMI, and smoking index, were collected retrospectively and compared between the 2 groups. Postoperative acute kidney injury refers to 1 of the following situations:

(1) serum creatinine increased by at least 0.3 mg/dL compared with the baseline within 48 hour;
(2) serum creatinine at least 1.5-fold higher than the baseline within 7 days; or
(3) urine volume ≦ 0.5 mL/kg/h lasting 6 hour.

### 2.2 Definitions

All definitions were based on criteria established by the Centers for Disease Control and Prevention. The most common types of NI are hospital-acquired pneumonia (HAP), ventilator-associated pneumonia (VAP), bloodstream infection (BSI), catheter-related bloodstream infection (CRBSI), catheter-associated urinary tract infection and surgical site infection (SSI).^[6–8]^ VAP is a subset of HAP that occurs more than 48 h after endotracheal intubation. In our study, both HAP and VAP were included into the type of pneumonia. CRBSI is a subset of BSI characterized by the presence of central venous catheters and signs of catheter insertion site infection. SSI can be divided into three types: superficial SSI, deep SSI and mediastinitis.

Antibiotic resistance means acquired resistance, except for natural resistance. Multidrug-resistant bacteria means that 1 bacteria was resistant to three or more classes of antibiotics, and bacteria sensitive only to colistin and/or tigecycline were defined as extensively drug resistant bacteria.^[9]^ Smoking index refers to the product of number of cigarettes per day and years of smoking. VIS score can be utilized to evaluate the circulation situation and usage of intropes, it equals dopamine (ug/kg·min) + dobutamine (ug/kg·min) + 10 × milrinone (ug/kg·min) + 100 × epinephrine (ug/kg·min) +100 × norepinephrine (ug/kg·min) + 10000 × pituitrin (ug/kg·min). Low cardiac output syndrome (LCOS) refers to the syndrome of reduced cardiac output (CO < 2.0L/min/m^2^) and peripheral malperfusion.

### 2.3 Data analysis

Continuous variables are shown as means plus SD; categorical data are presented as proportions. For comparisons of continuous variables, the t-test or Wilcoxon test was used, depending on the distribution of data. The categorical data were compared using the chi-square or Fisher exact test. Variables associated with the development of NI in the univariate analysis (p < 0.05) were included into a forward multivariable logistic regression model. All statistical analyses were performed using SPSS 21. A P value < 0.05 was considered statistically significant.

## 3 Results

### 3.1 Source of nosocomial infection and associated mortality

**Figure 1:**
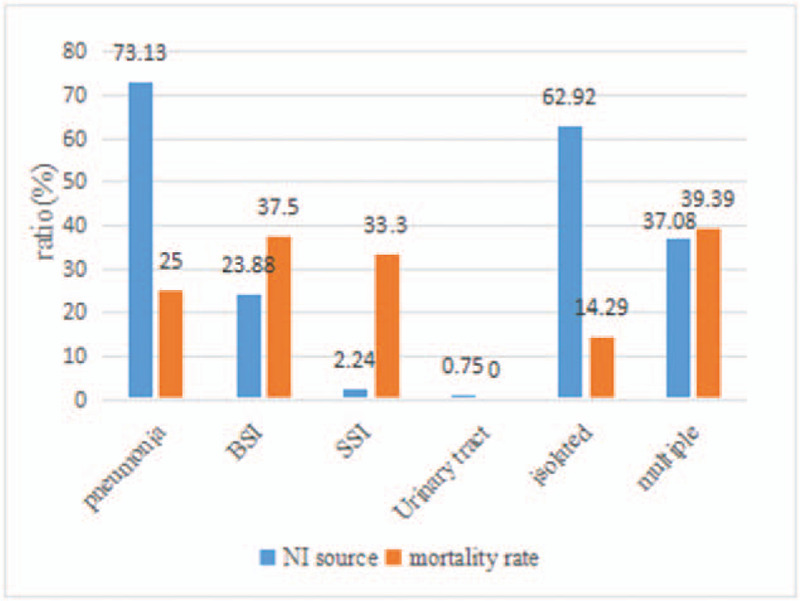
Source of NI and associated mortality.

**Figure 2:**
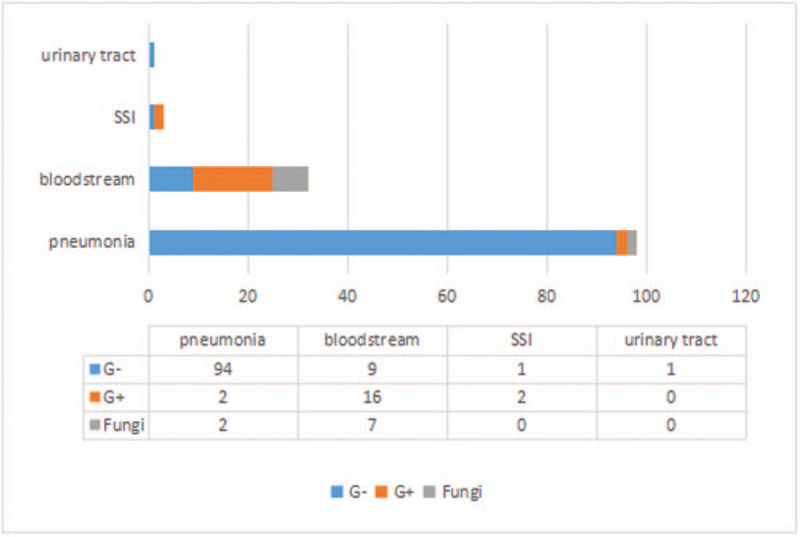
Distribution of pathogens in clinical specimens.

Out of 1360 patients enrolled into our study, 89 patients (6.54%) acquired a microbiologically documented NI. Among these 89 patients, 21 patients died, resulting in a mortality rate of 23.60%. For the other 1271 patients without NI, the mortality rate was only 2.28% (29/1271, p < 0.00). A total of 56 patients (62.92%) developed a single infection, while 33 patients (37.08%) experienced 2 or more different nosocomial infections. A total of 134 strains of pathogenic bacteria were detected, of which 98 (73.13%) were isolated from sputum and 32 (23.88%) were isolated from blood. Three (2.24%) SSIs were detected in our study (2 superficial SSIs and 1 mediastinitis). Only 1 (0.75%) bacterial strain was isolated from urine. The details of the sources of NI and associated mortality are shown in Figure 1. The distribution of pathogens for each type of NI were different. Microorganisms causing pneumonia were Gram-negative bacilli, while the main pathogens of BSI were Gram-positive cocci (Fig. 2).

### 3.2 Constituent ratio of pathogenic bacteria

A total of 134 strains of pathogenic bacteria were isolated in our study. The most common pathogens were Gram-negative bacteria (78.36%), followed by Gram-positive bacteria (14.93%). Table 1 presents the constituent ratio of predominant infectious pathogens.

**Table 1:** Constituent ratio of pathogenic bacteria.

Pathogenic Bacteria	Strains	Ratio (%)
Gram-negative	105	78.36
Acinetobacter baumannii	50	37.31
Klebsiella pneumoniae	19	14.18
Pseudomonas aeruginosa	17	12.69
Enterobacter cloacae	8	5.97
Escherichia coli	6	4.48
others	5	3.73
Gram-positive	20	14.93
Staphylococcus epidermidis	9	6.72
Enterococcus faecium	6	4.48
Staphylococcus aureus	3	2.24
others	2	1.49
Fungi	9	6.72
candida	8	5.97
aspergillus	1	0.75

### 3.3 Antibiotic resistance of main pathogens of NI in clinical practice

Along with the development and inappropriate use of antibiotics, especially wide-spectrum ones, drug resistance in bacteria changes constantly. Timely and continuous determination of antibiotic susceptibility is required. The details of antibiotic resistance for common pathogens are as follows (Tables 2 and 3).

**Table 2:** Resistant rates of common Gram-positive bacteria.

	Acinetobacter baumannii			Klebsiella pneumoniae			Pseudomonas aeruginosa			Enterobacter cloacae			Escherichia coli		
Antibiotics	tested strains	resistant strains	resistant rate (%)	tested strains	resistant strains	resistant rate (%)	tested strains	resistant strains	resistant rate (%)	tested strains	resistant strains	resistant rate (%)	tested strains	resistant strains	resistant rate (%)
Ampicillin	–	–	–	13	12	92.31	–	–	–	–	–	–	6	5	83.33
Piperacillin/Tazobactam	–	–	–	19	2	10.53	17	1	5.88	8	0	0	6	0	0
Ceftazidime	44	44	100.00	19	2	10.53	17	1	5.88	8	3	37.50	6	3	50.00
Ceftriaxone	50	45	90.00	19	5	26.32	–	–	–	8	3	37.50	6	3	50.00
Cefperazone-Sulbactam	50	17	34.00	–	–	–	17	1	5.88	-	–	–	–	–	–
Cefepime	50	44	88.00	19	2	10.53	17	1	5.88	8	3	37.50	6	2	33.33
Aztreonam	–	–	–	19	2	10.53	17	3	17.65	8	4	50.00	6	3	50.00
Gentamicin	50	44	88.00	19	2	10.53	17	2	11.76	8	0	0	6	3	50.00
Amikacin	7	7	100.00	19	2	10.53	17	1	5.88	8	0	0	6	0	0
Imipenem	48	44	91.67	19	2	10.53	17	5	29.41	8	0	0	6	0	0
Meropenem	47	42	89.36	19	2	10.53	17	1	5.88	8	0	0	6	0	0
Levofloxacin	50	21	42.00	19	2	10.53	17	1	5.88	8	1	12.50	6	5	83.33
Ciprofloxacin	50	45	90.00	19	2	10.53	17	1	5.88	8	1	12.50	6	5	83.33
Sulfamethoxazole	50	41	82.00	19	5	26.32	–	–	–	8	6	75.00	6	2	33.33
Minocycline	42	16	38.10	–	–	–	–	–	–	–	–	–	–	–	–
Tobramycin	50	42	84.00	19	2	10.53	17	1	5.88	8	0	0	6	0	0
Tigecycline	49	0	0	2	0	0	1	1	100	–	–	–	–	–	–

**Table 3:** Resistant rates of common Gram-positive bacteria.

	Staphylococcus epidermidis			Enterococcus faecium		
Antibiotics	tested strains	resistant strains	resistant rate (%)	tested strains	resistant strains	resistant rate (%)
Penicillin G	9	9	100	6	6	100
Oxacillin	9	8	88.89	6	6	100
High concentration of Gentamicin	9	7	77.78	6	6	100
Levofloxacin	9	6	66.67	6	6	100
Ciprofloxacin	9	6	66.67	6	6	100
Clindamycin	9	5	55.56	-	-	-
Rifampicin	9	2	22.22	6	3	50
Sulfamethoxazole	9	4	44.44	-	-	-
Vancomycin	9	0	0	6	1	16.67
Linezolid	9	0	0	6	0	0
Daptomycin	9	0	0	6	0	0

A total of 9 strains of fungi were isolated in our study, 8 of which were *Candida* species, 7 *C albicans* and 1 *C tropicalis*. All of the 7 *C albicans* isolates were susceptible to azoles, 5-fluorocytosine and amphotericin B. Aspergillosis was naturally resistant to fluconazole but sensitive to voriconazole and echinocandins.

### 3.4 Risk factors for NI

Table 4 shows the results of all potential variables associated with the development of NI. Eighteen of the 30 original variables, which had significant differences, were studied further using multivariate stepwise logistic regression analysis. The following six independent risk factors for the development of NI were identified in our study: duration of surgery, LCOS, continuous veno-venous hemofiltration, mechanical ventilation time, reintubation and tracheostomy (Table 5).

**Table 4:** Univariate analysis of perioperative risk factors of NI in patients after open heart surgery.

Variable	NI (n = 89)	non-NI (n = 1271)	*P*
Age (yr)	67.90 ± 11.76	61.83 ± 9.65	.002
Male	56 (62.9%)	740 (58.2%)	.425
BMI	27.35 ± 2.15	25.2 ± 3.56	.023
Smoking index	170.35 ± 101.48	152.00 ± 87.96	.760
Preoperative LVEF (%)	43 ± 11.32	47 ± 9.01	.001
Preoperative creatinine (umol/L)	78.50 ± 24.08	62.56 ± 26.50	.223
Preoperative albumine (g/L)	40.75 ± 3.86	41.01 ± 5.44	.923
History of COPD	9 (10.1%)	97 (7.6%)	.511
Diabetes mellitus	29 (32.6%)	365 (28.7%)	.595
Prior cancer treatment	2 (2.3%)	26 (2.1%)	.896
History of cerebrovascular disease	6 (6.7%)	77 (6.1%)	.698
History of immunosuppression	2 (2.2%)	11 (0.9%)	<.001
endocarditis	3 (3.4%)	40 (3.1%)	.899
Urgent Operation	10 (11.2%)	89 (7.0%)	.022
Type of operation			
CABG	18	479	.079
Valve	20	450	
Combination of CABG and valve	12	130	
Aortic root replacement	8	48	
Aortic arch surgery	31	165	
Duration of Surgery (min)	616.00 ± 88.47	525.83 ± 77.57	.019
CPB time (min)	277.50 ± 33.93	239.08 ± 45.61	.040
Aortic crossclamp time (min)	169.70 ± 15.13	151.25 ± 25.85	.061
Blood transfusion volume within 24h (mL)	654.37 ± 126.81	472.15 ± 154.93	.018
reoperation	3 (3.37%)	35 (2.75%)	.768
VIS score of intropes	347 ± 189.70	178 ± 202.15	.013
LCOS	29 (32.58%)	57 (4.48%)	<.001
Postoperative AKI	23 (29.2%)	76 (5.98%)	.002
CVVH	14 (15.73%)	25 (1.97%)	.001
Urgent insertion of IABP	8 (8.98%)	40 (3.15%)	.008
Mechanical ventilation time (h)	32.30 ± 22.1	8.67 ± 18.49	<.001
Reintubation	20 (22.47%)	71 (5.59%)	.001
Tracheostomy	18 (52.9%)	3 (4.5%)	<.001
ICU stay (d)	21 ± 17.69	4.68 ± 8.18	.012
Postoperative stroke	13 (14.61%)	50 (3.93%)	.006

AKI = acute kidney injury, COPD = chronic obstructive pulmonary disease, CPB = cardiopulmonary bypass, CVVH = continuous veno-venous hemofiltration, IABP = intra-aortic balloon pump, ICU = intensive care unit, LCOS = low cardiac output syndrome, LVEF = left ventricular ejection fraction.

**Table 5:** Multivariate stepwise logistic regression analysis of risk factors for the development of NI.

Variable	OR (95% CI)	*P*
duration of surgery	3.94 (1.07–10.04)	.04
LCOS	4.76 (1.43–15.72)	.01
CVVH	5.89 (1.27–16.12)	.008
mechanical ventilation time	15.24 (4.07–36.72)	.001
reintubation	21.09 (11.15–44.21)	.001
tracheostomy	12.38 (3.56–24.21)	.002

CI = confidence interval, CVVH = continuous veno-venous hemofiltration, LCOS = low cardiac output syndrome.

## 4 Discussion

In our retrospective study, HAP was the main cause of postoperative NI, accounting for 73.13% of cases. It was also a leading cause of mortality, as high as 17.34%. In accordance with our results, mechanical ventilation time and reintubation were independent risk factors for the development of NI. The development of HAP was associated with the use of endotracheal intubation and mechanical ventilation. Intubation destroys the normal barrier of the epiglottis and decreases the cough reflex and movement of cilia, which lead to impaired clearance of the infectious pathogen from airway secretions. Sputum was a good culture medium for bacteria. Mechanical ventilation also contributed to the development of VAP and the risk peaked within the first week. The initial step of VAP is the colonization of the upper respiratory tract by potentially pathogenic bacteria. Aspiration of these microorganisms either through the endotracheal tube or a leak around the cuff allowed them to enter the lower respiratory tract. This event, along with decreased host immunity, can result in the development of NI. The key to control the development of VAP is to reduce the duration of intubation, including the use of a semi-recumbent position, sedation holds, protocolized weaning and daily assessment for the possibility of extubation.

The predominant bacteria for HAP was *Acinetobacter baumannii*. *A baumannii* is a conditional pathogen that may cause NI in critically ill patients. *A baumannii* has simple growth requirements and may survive in a desiccated environment for prolonged periods.^[10]^ Contaminated environmental sources and transmission via medical personnel may cause outbreaks of NI.^[11,12]^*A baumannii* has been associated with high morbidity and mortality.^[13,14]^ Vincent JL and colleagues reported that infection with *A baumannii* was independently associated with a greater risk for hospital death among 14414 ICU patients.^[15]^ In recent years, the incidence and antibiotic resistance of *A baumannii* infection have increased rapidly. Treatment of *A baumannii* is difficult, owing to its resistance to various antibiotics and remarkable ability to acquire new resistance via different mechanisms, such as plasmids, transposons, integrons and resistance islands. Antimicrobial resistance poses a serious threat worldwide. In early 1990, carbapenem-resistant (CPR) strains of *A baumannii* were first discovered. CPR-*A baumannii* is often resistant to all classes of antibiotics, except for colistin and tigecycline.^[16]^ Furthermore, a large dose of sulbactam, fluoroquinolones, aminoglycosides and tetracyclines may also have antibacterial activity against CPR-*A baumannii*.^[17–20]^ In our study, we discovered that the carbapenem resistance rate of *A baumannii* had reached up to >50%. As much as 90% of *A baumannii* were sensitive to colistin and tigecycline, while the resistance rates to cefoperazone-sulbactam, levofloxacin and minocycline were 34.00%, 42.00% and 38.10%, respectively. We routinely analyzed the drug sensitivity of gentamicin, instead of amikacin, as a representative of aminoglycosides, and the resistance rate exceeded 80%.

The principles of treatment for *A baumannii* were as follows: 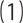 antimicrobial susceptibility results; 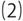 combination therapy; 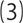 adequate dose; 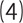 sufficient treatment period; and 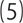 personal administration. Optimal therapy was established according to antimicrobial susceptibility results. However, for multidrug resistant or extensively drug resistant *A baumannii*, the recommended therapy was colistin/tigecycline combined with other agents (ie, carbapenem, sulbactams, fluoroquinolones or minocycline).

Except for *A baumannii*, *Klebsiella pneumoniae*, *Pseudomonas aeruginosa* and other Gram-negative bacilli were also common pathogens for HAP. As proved in our study, all of them were clinically sensitive to carbapenem, extended-spectrum cephalosporins and fluoroquinolones.

BSI is also a serious and common type of NI. It is more likely to occur in patients immunosuppressed and malnourished patients and those using various invasive devices. CRBSI is a subset of BSI with the presence of central venous catheters. Immediately after insertion, the catheter becomes coated with plasma protein. Bacteria could migrate from the skin along the surface of catheter. This may happen a few hours or more than 1 week after insertion. Femoral venous catheters have the highest rate of infection, followed by internal jugular and subclavian catheters.^[21]^ For CRBSI, once infection is suspected, the central catheter should be removed as soon as possible. In our research, the most common pathogens for BSI were Gram-positive cocci (50%), for example, *Staphylococcus* and *Enterococcus*. Within the past few decades, the resistance rate among Gram-positive cocci have obviously increased. We reported that 78.13% of Gram-positive bacteria were resistant to methicillin and only 6.25% were resistant to vancomycin. However, all of them were sensitive to linezolid.

Fungal infection is not unusual after cardiac surgery. Risk factors for fungal infection include immunosuppression, malnutrition, diabetes mellitus and long-term use of extended-spectrum antibiotics.^[22,23]^ In our research, *Candida* was the most common agent of fungal infection (88.89%; 8/9), which was consistent with previous studies.^[24–26]^ Among 8 strains of *Candida*, 6 were *C albicans* and 2 were *C. tropicalis*, with 7 strains isolated from the bloodstream. Other than *Candida*, Aspergillosis was also a common agent of fungal infection. Lung was the most frequent site of aspergillosis infection.^[27]^ Consistent with our results, most *Candida* infections, except for *C glabrata*, were susceptible to fluconazole and voriconazole. Aspergillosis was naturally resistant to fluconazole. Echinocandins were the best choice for definite and severe fungal infection, because they remained close to 100% effective.

As discovered in our research, the development of NI was associated with longer surgery duration, postoperative LCOS and utilization of continuous veno-venous hemofiltration. This finding suggested that NI may occur in patients with more severe diseases or more complex surgical procedures. For these populations, more attention should be paid to preoperative assessment and preparation and the choice of optimal surgical procedures and efforts should be made to minimize the duration of the surgery. Once postoperative LCOS or acute kidney injury has developed, sterile practices should be strictly followed for invasive operations, and a timely adjustment of the prescribed antibiotics according to the condition of the patient is crucial.

The distribution of pathogenic bacteria and antibiotic resistance of NI after cardiac surgery varies distinctly worldwide, and while our research data only provide the epidemiological profiles and trends of our institution, our results have significant implications for the prevention and treatment of NI.

## 5 Conclusion

NI is a common postoperative complication of open-heart surgery and is associated with increased morbidity and mortality. Currently, antimicrobial resistance, which differs in specific regions and/or populations, has become a great challenge for the treatment of NI. Therefore, we analyzed the characteristics of the distribution of pathogens, antibiotic resistance and independent risk factors for NI in patients after open-heart surgery and provided some suggestions for clinical practice. In addition to antibiotic treatment, avoidance of risk factors and aggressive infection control measures, including identifying the source of infection, environmental cleaning, contact precautions, isolation of infected patients and hand hygiene, may be crucial to stop or prevent outbreaks.

## Acknowledgments

In writing this paper, I have benefited from the input of my teachers and colleagues. They generously helped me collect materials I needed and made many invaluable suggestions. I hereby extend my gratitude for their kind help. Particularly, I am deeply indebted to Professor Qian Zhai, my supervisor, who guided me throughout my writing of this thesis. She carefully read the whole draft and offered painstaking and valuable feedback. Her standards of academic excellence have made my revision an exciting and gratifying experience. I also wish to sincerely thank my director, whose brilliant ideas and perceptive observations have proved immensely constructive. Furthermore, none of this would have been possible without the help of the following individuals and organizations: our department and its staff and the Department of Cardiovascular Surgery of Qilu Hospital of Shandong University and its staff.

## Author contributions

**Conceptualization:** zhengqin liu, xiquan zhang, Qian Zhai.

**Data curation:** zhengqin liu.

**Formal analysis:** zhengqin liu.

**Investigation:** zhengqin liu, Qian Zhai.

**Methodology:** zhengqin liu, xiquan zhang, Qian Zhai.

**Project administration:** zhengqin liu, xiquan zhang, Qian Zhai.

**Resources:** zhengqin liu.

**Software:** zhengqin liu.

**Supervision:** xiquan zhang, Qian Zhai.

**Validation:** zhengqin liu, Qian Zhai.

**Writing – original draft:** zhengqin liu.

**Writing – review & editing:** zhengqin liu, xiquan zhang, Qian Zhai.

## Abbreviations

Abbreviations: BSI = blood stream infection, CPR = carbapenem resistant, CRBSI = catheter-related blood stream infection, HAP = hospital-acquired pneumonia, ICU = intensive care unit, LCOS = low cardiac output syndrome, LVEF = left ventricular ejection fraction, NI = nosocomial infections, SSI = surgical site infection, VAP = ventilator-associated pneumonia.
